# Evaluation of Two Liver Treatment Strategies in a Mouse Model of Niemann–Pick-Disease Type C1

**DOI:** 10.3390/ijms19040972

**Published:** 2018-03-24

**Authors:** Lynn Ebner, Anne Gläser, Anja Bräuer, Martin Witt, Andreas Wree, Arndt Rolfs, Marcus Frank, Brigitte Vollmar, Angela Kuhla

**Affiliations:** 1 Institute for Experimental Surgery, Rostock University Medical Center, Schillingallee 69a, 18057 Rostock, Germany; LynnEbner@gmx.de (L.E.); brigitte.vollmar@med.uni-rostock.de (B.V.); 2 Institute of Anatomy, Rostock University Medical Center, Gertrudenstraße 9, 18057 Rostock, Germany; anne.glaeser@uni-oldenburg.de (A.G.); AnjaUrsula.Braeuer@med.uni-rostock.de (A.B.); martin.witt@med.uni-rostock.de (M.W.); andreas.wree@med.uni-rostock.de (A.W.); 3 Albrecht Kossel Institute for Neuroregeneration, Rostock University Medical Center, Gehlsheimer Straße 20, 18147 Rostock, Germany; arndt.rolfs@med.uni-rostock.de; 4 Medical Biology and Electron Microscopy Center, Rostock University Medical Center, 18057 Rostock, Germany; marcus.frank@med.uni-rostock.de

**Keywords:** NPC1 mutant, liver dysfunction, miglustat, allopregnanolone, cyclodextrin

## Abstract

Niemann–Pick-disease type C1 (NPC1) is an autosomal-recessive cholesterol-storage disorder. Besides other symptoms, NPC1 patients develop liver dysfunction and hepatosplenomegaly. The mechanisms of hepatomegaly and alterations of lipid metabolism-related genes in NPC1 disease are still poorly understood. Here, we used an NPC1 mouse model to study an additive hepatoprotective effect of a combination of 2-hydroxypropyl-β-cyclodextrin (HPβCD), miglustat and allopregnanolone (combination therapy) with the previously established monotherapy using HPβCD. We examined transgene effects as well as treatment effects on liver morphology and hepatic lipid metabolism, focusing on hepatic cholesterol transporter genes. Livers of *Npc1*^−/−^ mice showed hepatic cholesterol sequestration with consecutive liver injury, an increase of lipogenetic gene expression, e.g., *HMG-CoA*, a decrease of lipolytic gene expression, e.g., *pparα* and *acox1*, and a decrease of lipid transporter gene expression, e.g., *acat1*, *abca1* and *fatp2*. Both, combination therapy and monotherapy, led to a reduction of hepatic lipids and an amelioration of NPC1 liver disease symptoms. Monotherapy effects were related to *pparα*- and *acox1-*associated lipolysis/β-oxidation and to *fatp2*-induced fatty acid transport, whereas the combination therapy additionally increased the cholesterol transport via *abca1* and *apoE*. However, HPβCD monotherapy additionally increased cholesterol synthesis as indicated by a marked increase of the *HMG-CoA* and *srebp-2* mRNA expression, probably as a result of increased hepatocellular proliferation.

## 1. Introduction

Niemann–Pick-disease type C1 (NPC1) is an autosomal-recessive lipid-storage disorder characterized by neonatal jaundice, hepatosplenomegaly, and progressive neurodegeneration [1,2]. The mutation responsible for approximately 95% of these cases has been mapped to a gene on chromosome 18q11 designated *NPC1* [3]. In the absence of NPC1 function, unesterified cholesterol and other lipids accumulate in the endosome/lysosome (LE/L) in NPC-mutant cells, where it is inaccessible to the sterol-sensing machinery in the cytosol and nucleus [4]. One gene, which utilizes long-chain fatty acyl-coenzyme A (CoA) and cholesterol as substrates to form cholesteryl esters, is acyl-CoA cholesterol acyltransferase (*acat1*) [5]. This enzyme has been shown to recover cholesterol esterification in macrophages with an NPC1 phenotype [6]. Several other studies using the BALB/cJ *Npc1* mouse model have demonstrated that the transport of lipoprotein-derived lipids (cholesterol and free fatty acids) from late LE/L to other cellular compartments is disturbed [7,8,9]. Thereby, cholesterol transport fails to maintain cellular, tissue, and whole-body lipid homeostasis. The lipid metabolism in NPC1 disease also fails through feedback inhibition of the sterol regulatory element-binding protein (SREBP) pathway [10]. Beside the SREBP pathway, the peroxisome proliferator-activated receptor (PPAR) pathway with the genes peroxisomal acyl-coenzyme A oxidase 1 (*acox1*) and fatty acid transport protein 2 (*fatp2*) is implicated in the regulation of the free fatty acid hepatic metabolism. Moreover, *pparα* regulates the expression of liver genes involved in mitochondrial and/or peroxisomal fatty acid β-oxidation, and *pparα* gene expression is decreased in livers of C57BL/6J *Npc1* mutant mice [10]. Therefore, decreased expression of the *pparα* gene provides a plausible explanation for accumulation of free fatty acids in NPC1 disease. Target genes of *pparα* are apolipoprotein E (*apoE*) and ATP-binding cassette A1 (*abca1*), which play a role in transporting cholesterol to the plasma membrane [11]. Furthermore, in vitro studies showed that the relative amount of *abca1* mRNA and protein was decreased in human and mouse NPC1-defective cells [12,13].

The iminosugar miglustat (Zavesca^®^, Actelion Pharmaceuticals, Allschwil, Switzerland) is currently the only disease-modifying therapy approved for NPC1 disease in Europe [14,15]. Miglustat is a small molecule, which inhibits the glycosylceramid synthase, one of the key components of the glycosphingolipid biosynthesis, and thus reduces intracellular lipid storage [16]. Long-term therapy with miglustat has been shown to increase lifespan and stabilize neurologic functions. In addition, miglustat is believed to reduce oxidative stress [17]. Another promising drug is 2-hydroxypropyl-β-cyclodextrin (HPβCD)—a cyclic oligosaccharide. It is used as an enabling excipient in pharmaceutical formulations as well as a cholesterol modifier in vivo. HPβCD overcomes the transport defect leading to excretion of accumulated cholesterol as bile acid as shown in *Npc1^−/−^* mice [18]. It has been suggested that cholesterol efflux is mediated by ATP binding cassette subfamily G member 1 (ABCG1), which promotes biliary excretion of sterols ameliorating liver function [18,19]. In combination with miglustat and the neurosteroid allopregnanolone (ALLO), HPβCD reduced cerebellar neurodegeneration and improved motor function in *Npc1^−/−^* mice [20,21,22]. In addition, some reports have shown that already HPβCD alone improved cholesterol sequestration in organs and prolonged the lifespan of *Npc1^−/−^* mice [18,19,22,23]. In line with these findings, a clinical trail [24] reported that HPβCD was effective in NPC1 patients, suggesting HPβCD as a promising drug candidate for NPC1 disease.

In the present study, we aimed to investigate the role of cholesterol or fatty acid transporter, of lipolytic genes and genes of β-oxidation, e.g., *pparα*, *acox1*, *acat1*, *abca1*, *apoE* and *fat2p* for the manifestation of hepatomegaly and alterations of lipid metabolism in *Npc1^−/−^* mice. In addition, we examined the effect of HPβCD monotherapy compared with combination therapy with HPβCD/ALLO/miglustat on liver morphology and the hepatic lipid metabolism in this mouse model.

## 2. Results

Beneficial effects by combination therapy and monotherapy on liver morphology and liver function in *Npc1^−/−^* mice.

### 2.1. Body Weight

**Figure 1:**
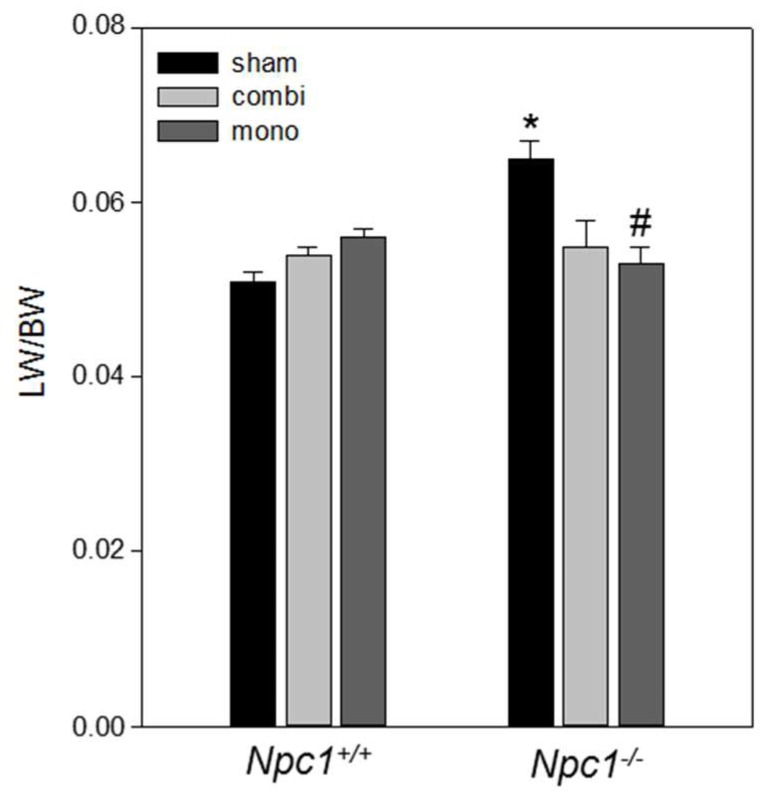
Evaluation of liver-to-body-weight ratios (LW/BW) of sham-treated, combination (combi)-treated and mono (HPβCD)-treated *Npc1^+/+^* (sham, *n* = 16; combi, *n* = 18; mono, *n* = 15) and *Npc1^−/−^* mice (sham, *n* = 16; combi, *n* = 11; mono, *n* = 11). Note the decrease of LW/BW ratio of mono-treated and combi-treated *Npc1^−/−^* mice. Values are given as mean ± SEM; ANOVA; *post-hoc* pairwise comparison tests: * *p* < 0.05 vs. sham-treated *Npc1^+/+^*; ^#^ *p* < 0.05 vs. sham-treated *Npc1^−/−^*.

Analysis of the liver to body weight (LW/BW) ratio showed that sham-treated *Npc1^−/−^* mice had a 1.3-fold increase of LW/BW ratio when compared to sham-treated *Npc1^+/+^* mice (Figure 1; *p* < 0.05), while both combination therapy and monotherapy (*p* < 0.05) markedly decreased LW/BW ratio and reached values found in sham-treated *Npc1^+/+^* mice (Figure 1).

### 2.2. Histology

**Figure 2:**
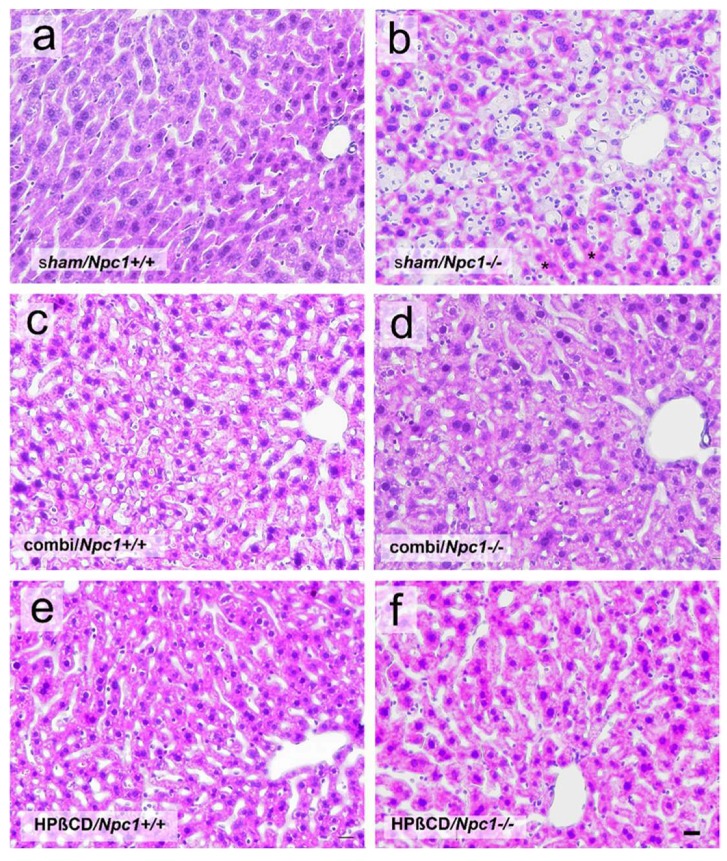
Hematoxylin & Eosin (H&E)-stained images of liver tissue of a sham-treated, a combination (combi)-treated and a mono (HPβCD)-treated *Npc1^+/+^* (**a**,**c**,**e**) and *Npc1^−/−^* mouse (**b**,**d**,**f**). Note the necrosis in the liver tissue of a sham-treated *Npc1^−/−^* mouse (asterisks, **b**). A scale bar is shown in **f** and also applies to **a**–**e**: 20 µm.

**Figure 3:**
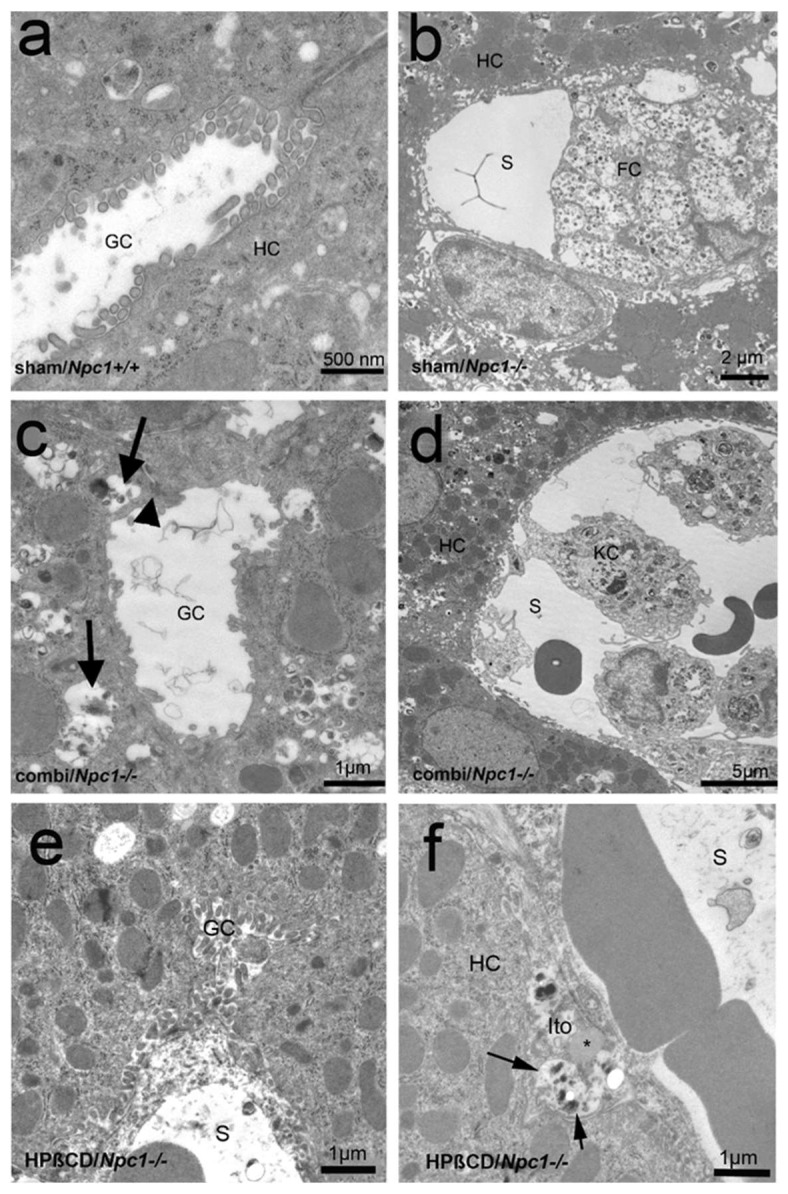
Electron micrographs of liver tissue of a sham-treated *Npc1^+/+^* (**a**) and *Npc1^−/−^* (**b**) mouse as well as of a combination (combi)-treated *Npc1^−/−^* (**c**,**d**) and a mono (HPβCD)-treated *Npc1^−/−^* (**e**,**f**) mouse. (**a**) Regular morphology of hepatocytes (HC) forming a gall capillary (GC). (**b**) A histiocytic foam cell (FC) congests the lumen of a hepatic sinusoid (S), whereas hepatocytes (HC) are only partly loaded with myelin-like material. (**c**,**f**), Despite combination therapy as well as monotherapy, there are still numerous inclusions in extended hepatocellular endoplasmic reticulum (ER) cisterns (bold (**c**) and regular arrows (**f**)) with some Ito cells in the neighborhood (**c**). Tight junctions (bold arrowhead) and mitochondria exhibit normal morphology. (**d**) Same specimen as (**c**) hepatic sinusoid with some von Kupffer cells (KC) whose extensions attach the sinusoidal endothelium. The cells contain typical myelin-like material, but there are no histiocytic foam cells that congest the sinusoidal lumen.

Hematoxylin and eosin (H&E) staining from sham-treated, combi-treated or mono-treated *Npc1^+/+^* mice exhibited regular liver morphology and normal microvascular integration (Figure 2a,c,e). In contrast, liver tissue of sham-treated *Npc1^−/−^* mice showed necrosis (Figure 2b). Further, liver architecture was characterized by lipid accumulation in hepatocytes (Figure 2b and Figure 3b) and, more common, invasion of histiocytic foam cells in sinusoids (Figure 2b and Figure 3b). Following combination therapy as well as monotherapy *Npc1^−/−^* mice showed an amelioration of liver morphology and less necrosis, but still some fatty deposits (Figure 2d,f).

### 2.3. Electron Microscopy

Electron microscopy of liver tissue revealed regular peribiliary microanatomy in sham-treated *Npc1^+/+^* animals (Figure 3a), whereas large and numerous myelin-like inclusions were detected in extended endoplasmic reticulum cisterns of sham-treated *Npc1^−/−^* hepatocytes as well as in sinusoidal foam cells, partly congesting the sinusoids (Figure 3b). Following long-term combination therapy and monotherapy, peribiliary inclusions were visible in *Npc1^−/−^* mice indicating that treatment did not completely abolish accumulation of lipid components (Figure 3c–f). Furthermore, single myelin-like inclusions, in macrophages, possibly von Kupffer cells (Figure 3d) or Ito cells (Figure 3f), were still visible, however, congestion of sinusoids was not observed anymore (Figure 3d).

### 2.4. Biochemistry

**Figure 4:**
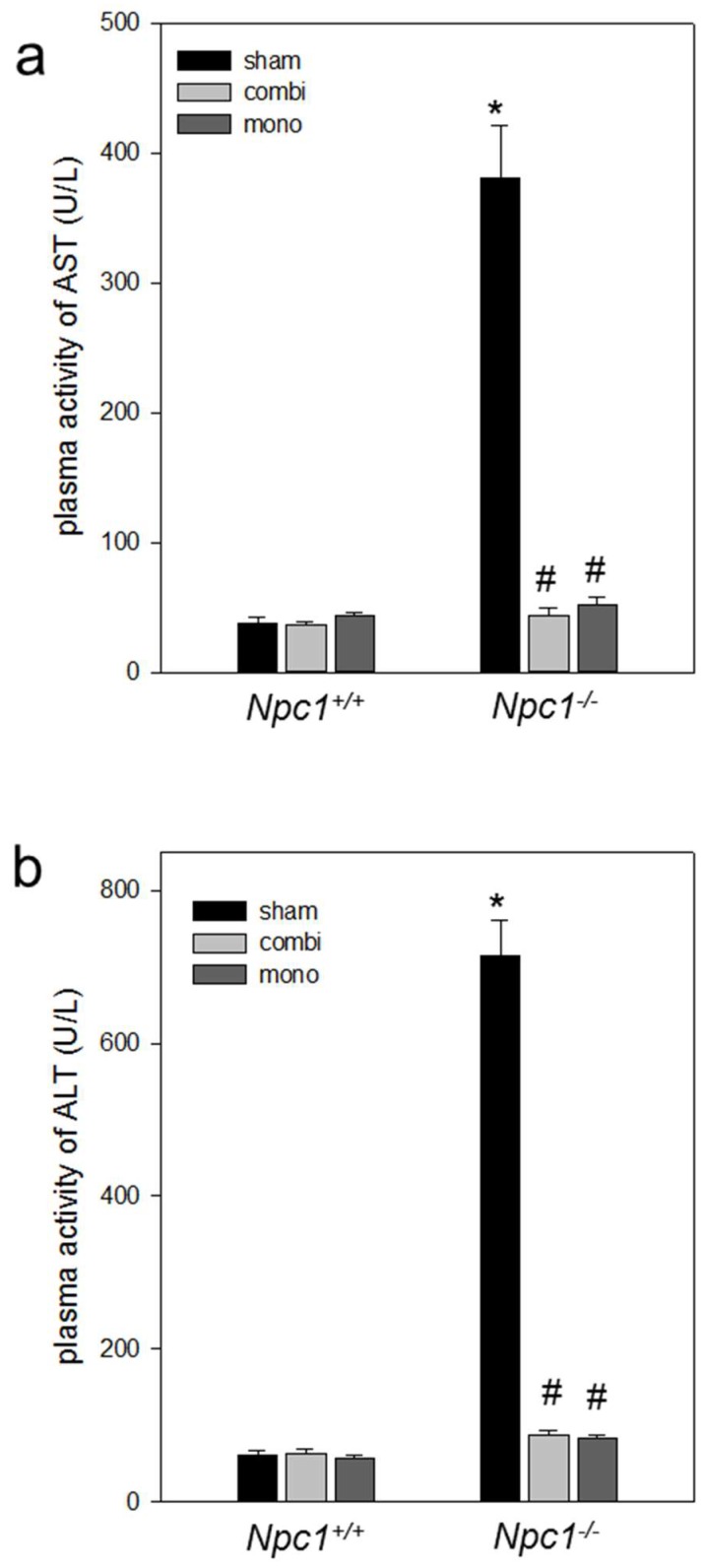
Analysis of plasma activities of aspartate aminotransferase (AST, **a**) and alanine aminotransferase (ALT, **b**) of sham-treated, of combination (combi)-treated as well as of mono (HPβCD)-treated *Npc1^+/+^* (sham, *n* = 16; combi, *n* = 18; mono, *n* = 15) and *Npc1^−/−^* mice (sham, *n* = 16; combi, *n* = 11; mono, *n* = 11). Note the amelioration of liver transaminases in combi-treated and mono-treated *Npc1^−/−^* mice. Values are given as mean ± SEM; ANOVA; *post-hoc* pairwise comparison tests: * *p* < 0.05 vs. sham-treated *Npc1^+/+^*; ^#^ *p* < 0.05 vs. sham-treated *Npc1^−/−^*.

Measurements of aspartate aminotransferase (AST) and alanine aminotransferase (ALT) plasma activities in *Npc1^+/+^* mice with and without combination therapy as well as monotherapy presented standard values of AST (~40 U/L, Figure 4a) and ALT (~60 U/L, Figure 4b). Significantly increased AST (*p* < 0.05) and ALT (*p* < 0.05) levels were determined in the plasma of sham-treated *Npc1^−/−^* mice when compared to *Npc1^+/+^* mice. Both the combination therapy and the monotherapy reduced the AST and ALT levels significantly (*p* < 0.05) and reached values similar to those found in sham-treated *Npc1^+/+^* mice (Figure 4).

### 2.5. Immunohistochemistry of Cell Dynamics—Apoptosis and Proliferation

**Figure 5:**
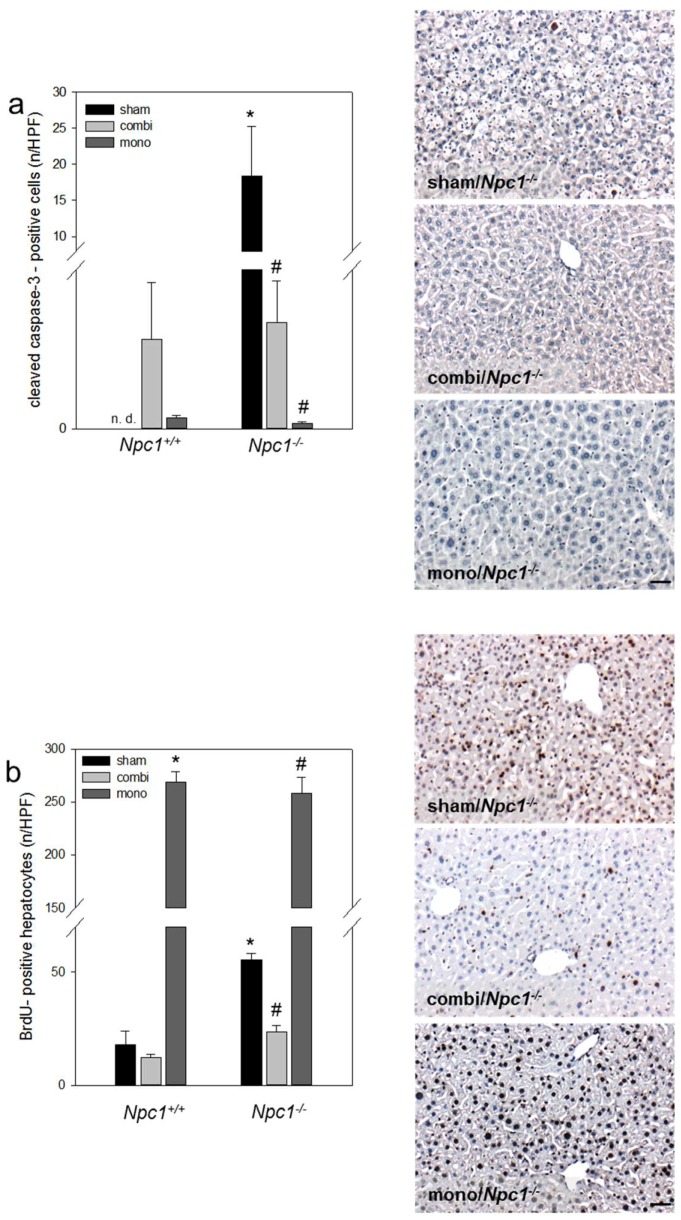
Quantitative analysis of cleaved caspase-3^+^ cells per high power field (HPF) (**a**) and of BrdU^+^ hepatocytes per HPF (**b**) in liver tissue of sham-treated, of combination (combi)-treated as well as of mono (HPβCD)-treated *Npc1^+/+^* (sham, *n* = 7 (BrdU *n* = 3); combi, *n* = 9 (BrdU *n* = 3); mono, *n* = 15 (BrdU *n* = 3) and *Npc1^−/−^* mice (sham, *n* = 10 (BrdU *n* = 3); combi, *n* = 10 (BrdU *n* = 3); mono, *n* = 11 (BrdU *n* = 3)) with representative light microscopic images of liver tissue of sham-treated, combi-treated and mono-treated *Npc1^−/−^* mice. Note the significant reduction of apoptosis and proliferation in combi-treated *Npc1^−/−^* mice. Values are given as mean ± SEM; ANOVA; *post-hoc* pairwise comparison tests: * *p* < 0.05 vs sham-treated *Npc1^+/+^*; ^#^ *p* < 0.05 vs. sham-treated *Npc1^−/−^*. Scale bar in **a** and **b**: 20 µm.

Combination therapy in *Npc1^−/−^* mice reduced hepatic apoptosis and proliferation while monotherapy reduced apoptosis but increased proliferation. Immunohistochemistry revealed, beside signs of necrosis, that *NPC1^−/−^* was also characterized by apoptotic cell death as shown by significantly increased numbers of cleaved caspase-3^+^ liver cells (*p* < 0.05) in sham-treated *Npc1^−/−^* vs. *Npc1^+/+^* mice (Figure 5a). Both the combination therapy and monotherapy significantly reduced (*p* < 0.05) the number of cleaved caspase-3^+^ cells (Figure 5a) in *Npc1^−/−^* mice. In addition, there was an increase of cell proliferation in sham-treated *Npc1^−/−^* vs. *Npc1^+/+^* mice as indicated by a significant rise of Bromodeoxyuridine (BrdU)^+^ hepatocytes (*p* < 0.017), which was, in turn, significantly reduced upon combination therapy in *Npc1^−/−^* mice (*p* = 0.018; Figure 5b). In contrast, monotherapy significantly increased the number of BrdU^+^ hepatocytes (*p* < 0.001; Figure 5b) in both mouse strains.

### 2.6. Inflammation and Cholesterol Homeostasis

#### 2.6.1. Both Combination Therapy and Monotherapy Reduced Hepatic Inflammation

We evaluated the infiltration of granulocytes (chloracetate esterase (CAE) staining) and macrophages (F4/80 reactivity) to clarify whether accumulation of lipids caused an inflammation. Sham-treated *Npc1^−/−^* mice revealed an almost three-fold increase of granulocytes when compared to sham-treated (44 ± 9 CAE^+^ cells vs. 15 ± 10 cells per high power field (HPF)).This effect was reduced upon combination therapy to 25 ± 5 CAE^+^ cells per HPF and upon monotherapy to 5 ± 1 CAE^+^ cells per HPF. However, F4/80^+^ macrophages showed no differences between *Npc1^−/−^* and *Npc1^+/+^* mice (4.5 ± 2 (*Npc1^+/+^*) vs. 4.0 ± 3 (*Npc1^−/−^*) cells per HPF). Likewise, upon combination therapy and monotherapy both mouse groups exhibited equal values which were markedly increased by up to 18 ± 6 (combi) and up to 12 ± 3 (mono) F4/80^+^ cells per HPF.

#### 2.6.2. Combination Therapy and In Particular Monotherapy Restored the Cholesterol Homeostasis in *Npc1^−/−^* Mice

**Figure 6:**
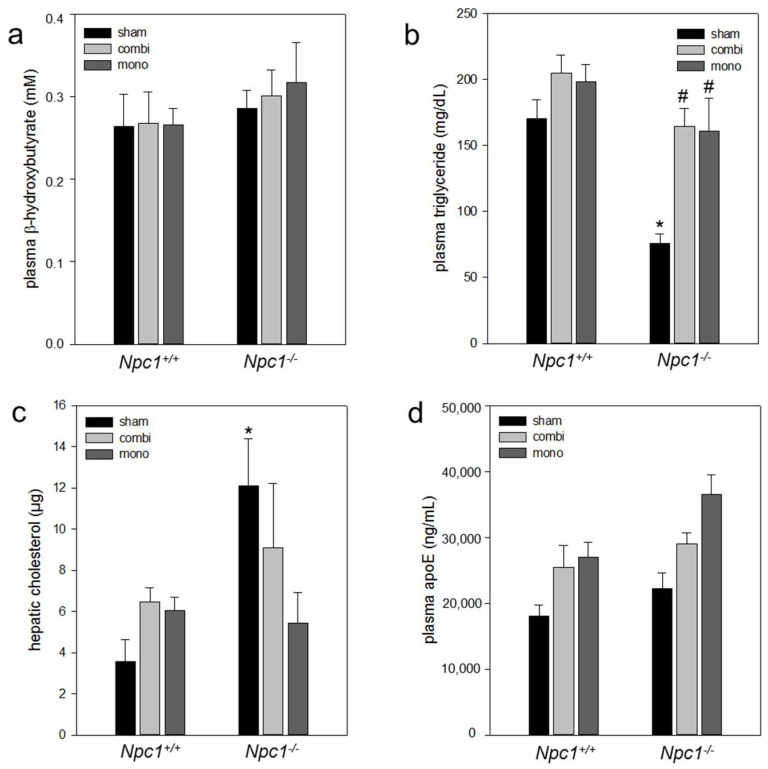
Analysis of plasma concentrations of β-hydroxybuyrate (**a**), triglycerides (**b**), hepatic cholesterol content (**c**) and of apoE (**d**) of sham-treated, of combination (combi)-treated as well as of mono (HPβCD)-treated *Npc1^+/+^* (sham, *n* = 16; combi, *n* = 18; mono, *n* = 15) and *Npc1^−/−^* mice (sham, *n* = 16; combi, *n* = 11; mono, *n* = 11). Note the significant reduction of triglycerides in sham-treated *Npc1^−/−^* vs. *Npc1^+/+^* mice which was significant increase upon combination therapy as well as monotherapy. Values are given as mean ± SEM; ANOVA; *post-hoc* pairwise comparison tests: * *p* < 0.05 vs. sham-treated *Npc1^+/+^*; ^#^ *p* < 0.05 vs. sham-treated *Npc1^−/−^*.

Since *Npc1^−/−^* mice exhibited a negative energy balance, increased ketogenesis can be assumed as an alternative energy source. The measurement of plasma β-hydroxybutyrate concentrations, a parameter reflecting ketogenesis, revealed no difference, neither between treatments nor between groups (Figure 6a). In addition, we examined the amount of systemic triglyceride concentrations, which showed a significant decrease (*p* < 0.001) in sham-treated *Npc1^−/−^* vs. *Npc1^+/+^* mice. Both combination therapy and monotherapy resulted in a significant increase (*p* = 0.001) of plasma triglyceride concentration in *Npc1^−/−^* mice when compared to sham-treated *Npc1^−/−^* mice (Figure 6b). The amount of hepatic cholesterol was significantly (*p* < 0.05) increased in sham-treated *Npc1^−/−^* vs. *Npc1^+/+^* mice (Figure 6c). The combination therapy slightly reduced the hepatic cholesterol content from 12.1 to 9.1 µg (Figure 6c). Monotherapy caused a two-fold decrease of cholesterol in *Npc1^−/−^* mice when compared to sham-treated *Npc1^−/−^* mice (Figure 6c). While the systemic concentration of the cholesterol transporter apoE showed equal values in both mouse strains, the combination as well as mono treatment revealed an up to 1.6-fold increase of plasma apoE concentrations in both *Npc1^−/−^* and *Npc1^+/+^* mice (Figure 6d).

**Figure 7:**
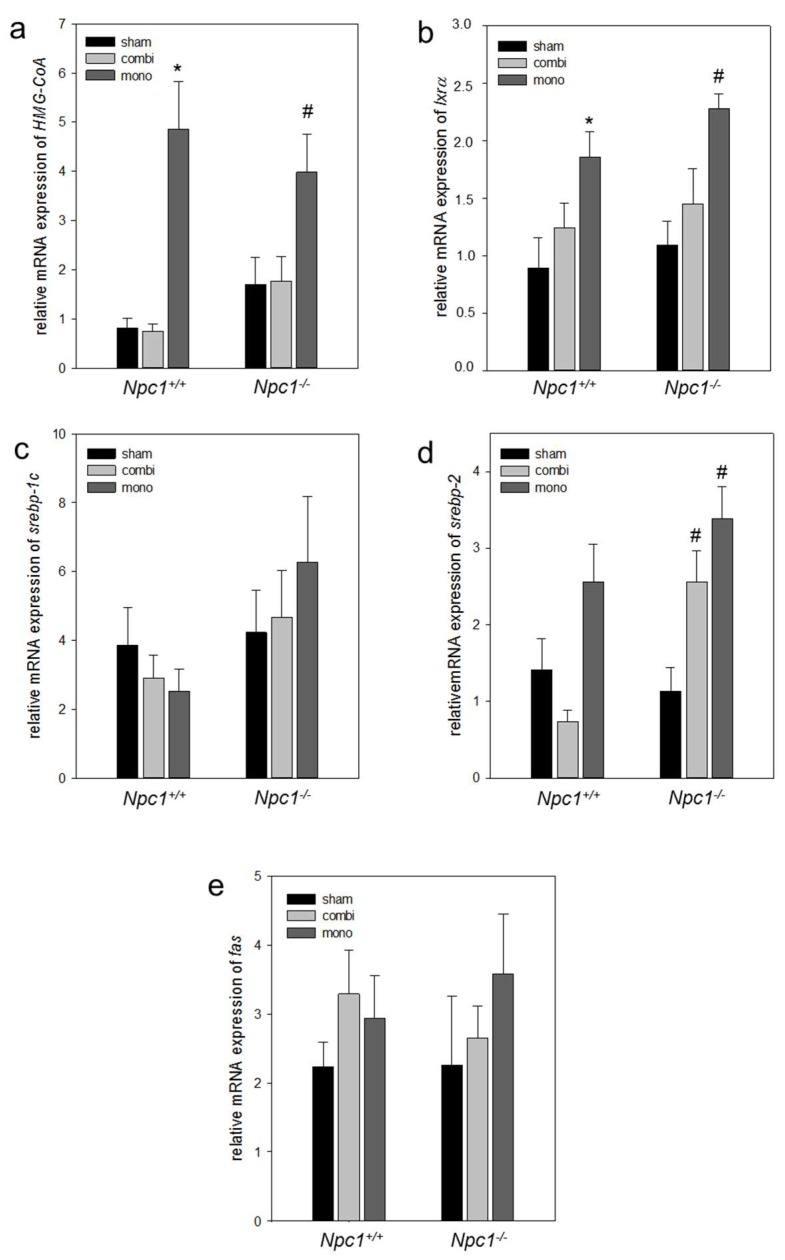
Quantitative real-time PCR analysis of *HMG-CoA* (**a**), *lxrα* (**b**), *srebp-1c* (**c**), *srebp-2* (**d**) and *fas* (**e**) in liver tissue of sham-treated, of combination (combi)-treated as well as of mono (HPβCD)-treated wild type (*Npc1^+/+^*, sham, *n* = 7; combi, *n* = 9; mono, *n* = 15) and *Npc1^−/−^* (sham, *n* = 10; combi, *n* = 10; mono, *n* = 11). Values are given as mean ± SEM; ANOVA; *post-hoc* pairwise comparison tests: * *p* < 0.05 vs. sham-treated *Npc1^+/+^*; ^#^ *p* < 0.05 vs. sham-treated *Npc1^−/−^*.

**Figure 8:**
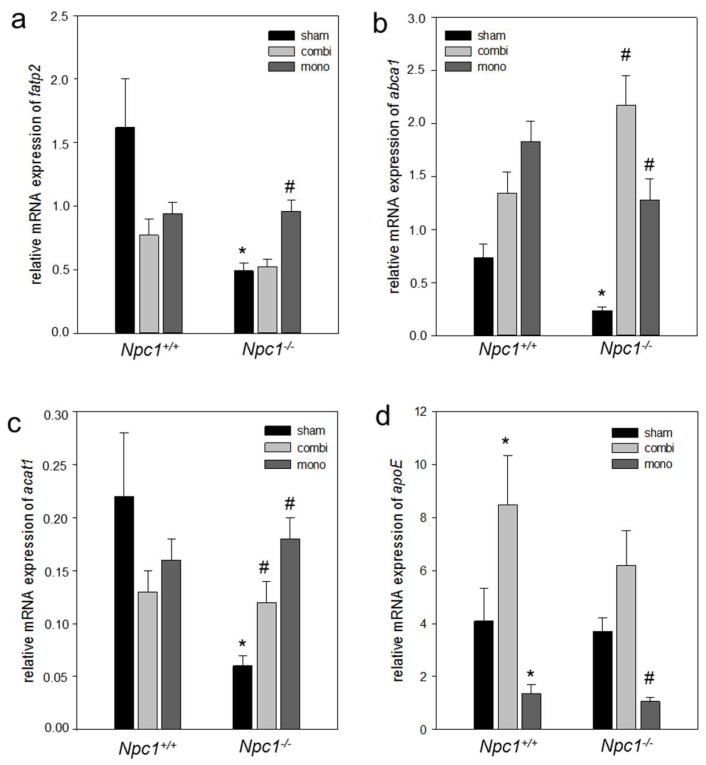
Quantitative real-time PCR analysis of *fatp2* (**a**), *abca1* (**b**), *acat1* (**c**) and *apoE* (**d**) in liver tissue of sham-treated, of combination (combi)-treated as well as of mono (HPβCD)-treated wild type (*Npc1^+/+^,* sham, *n* = 7; combi, *n* = 9; mono, *n* = 15) and *Npc1^−/−^* (sham, *n* = 10; combi, *n* = 10; mono, *n* = 11). Values are given as mean ± SEM; ANOVA; *post-hoc* pairwise comparison tests: * *p* < 0.05 vs. sham-treated *Npc1^+/+^*; ^#^ *p* < 0.05 vs. sham-treated *Npc1^−/−^*.

**Figure 9:**
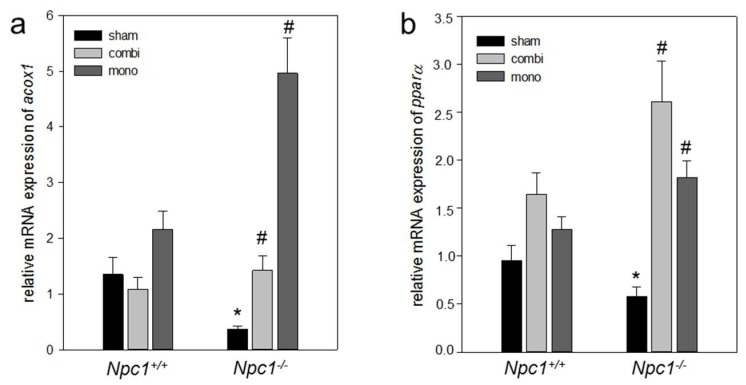
Quantitative real-time PCR analysis of *acox1* (**a**) and *pparα* (**b**) in liver tissue of sham-treated, of combination (combi)-treated as well as of mono (HPβCD)-treated wild type (*Npc1^+/+^*, sham, *n* = 7; combi, *n* = 9; mono, *n* = 15) and *Npc1^−/−^* (sham, *n* = 10; combi, *n* = 10; mono, *n* = 11). Values are given as mean ± SEM; ANOVA; *post-hoc* pairwise comparison tests: * *p* < 0.05 vs. sham-treated *Npc1^+/+^*; ^#^ *p* < 0.05 vs. sham-treated *Npc1^−/−^*.

In line with increased lipogenesis in sham-treated *Npc1^−/−^* mice, also the mRNA expression of *HMG-CoA* was two-fold more (Figure 7a) when compared to sham-treated *Npc1^+/+^* mice. However, the gene expressions of *lxrα*, *srebp-1c*, *srebp-2* and *fatty acid synthase* (*fas*) remained unchanged in both sham-treated *Npc1^+/+^* and *Npc1^−/−^* mice (Figure 7b–e). Upon combination therapy in *Npc1^−/−^* mice, the gene expression of *srebp-2* was significant (*p* = 0.023) when compared to sham-treated *Npc1^−/−^* mice (Figure 7d). In line with this, monotherapy also caused a significant increase (*p* < 0.05) of the *srebp-2* mRNA expression when compared to sham-treated *Npc1^−/−^* mice (Figure 7d). While gene expression of *fas*, *srebp-1c*, *lxrα*, and *HMG-CoA* were nearly unchanged upon combination therapy, monotherapy resulted in a significant increase of the *HMG-CoA* (*p* > 0.05) and *lxrα* (*p* > 0.05) mRNA expression (Figure 7a,b) indicating an increase of de novo lipogenesis. In addition, mRNA expression of lipid transporter genes such as *fatp2* (*p* < 0.05) and *abca1* (*p* = 0.001) was significantly decreased in *Npc1^−/−^* vs. *Npc1^+/+^* mice (Figure 8a,b) indicating a reduction of lipid transport in case of absent NPC function. Similarly, gene expression of *acat1* was significantly decreased (*p* < 0.05) in *Npc1^−/−^* vs. *Npc1^+/+^* mice (Figure 8a,c). However, the *apoE* mRNA expression was almost unchanged in *Npc1^−/−^* mice vs. sham-treated *Npc1^−/−^* mice (Figure 8d). Upon combination therapy and monotherapy in *Npc1^−/−^* the increase of gene expressions of *abca1* (*p* < 0.05) and *acat1* (*p* < 0.05) was significant (Figure 8b,c), which was more pronounced upon combination therapy indicating an intensified cholesterol transport. Otherwise, the mRNA expression *fatp2*, was significantly (*p* < 0.05) increased exclusively upon monotherapy (Figure 8a). While the mRNA expression of *apoE* was two-fold more upon combination therapy in *Npc1^−/−^* mice, monotherapy resulted in a significant (*p* < 0.05) decrease of *apoE* mRNA expression (Figure 8d). The expressions of lipolytic genes as well as of β-oxidation, i.e., of *pparα* (*p* = 0.021) and *acox1* (*p* = 0.003) in sham-treated *Npc1^−/−^* vs. *Npc1^+/+^* mice was significantly decreased (Figure 9). Of utmost interest, the combination therapy as well as the monotherapy significantly raised *pparα* (*p* < 0.05) and *acox1* (*p* < 0.05) mRNA expressions in *Npc1^−/−^* mice vs. sham-treated *Npc1^−/−^* mice. The increase of mRNA expression of *pparα* was more pronounced upon combination treatment indicating an intensified lipolysis (Figure 9b). However, the mRNA expression of *acox1* upon monotherapy was more upregulated in comparison to combination therapy (Figure 9a) indicating an intensified β-oxidation.

## 3. Discussion

In the absence of NPC1 function, unesterified cholesterol and other lipids accumulate in the LE/L in NPC-mutant cells, where it is inaccessible to the sterol-sensing machinery in the cytosol and nucleus [4]. While there is an excess of cholesterol trapped in the LE/L in NPC-mutant cells, these cells are in a state of cholesterol deprivation and therefore upregulate de novo cholesterol synthesis [4]. Consistent with previous studies [1,8,19] we confirmed an increase of hepatic cholesterol in *Npc1^−/−^* mice, which was accompanied by a decrease of plasma triglyceride concentrations. According to the knowledge that unesterified cholesterol accumulates in NPC disease, the current study showed a downregulation of mRNA expression of *acat1*, which utilizes long-chain fatty acyl-CoA and cholesterol as substrates to form cholesteryl esters [5]. Liver failure due to high cholesterol content was characterized by apoptotic cell death. This is in agreement with other previous studies [8,25] that showed an increased number of TdT-mediated dUTP-biotin nick end labeling (TUNEL)-stained hepatocytes. Furthermore, in accordance to Sayre et al. [25], we found that *Npc1^−/−^* mice exhibited strong hepatic proliferation. A link between pathologic hepatic lipid accumulation and stronger proliferation seems reasonable, for example based on the notion that after a partial hepatectomy, the liver stores fat to provide an energy pool for regeneration [26,27]. Additionally, the excessive accumulation of lipids in *Npc1^−/−^* mice was associated with hepatocellular necrosis and enhanced hepatic inflammatory activity, a widely known phenomenon of non-alcoholic fatty liver disease [28,29]. Thus, an NPC1 mutation causes steatohepatitis.

The master regulator of de novo cholesterol synthesis, the liver-X receptor α (*lxrα*), controls the expression of several lipogenetic genes such as *srebp-1c*, *fas* and *HMG-CoA* [30,31]. In support of this and in agreement with a previous study [32], we found that in case of NPC1 loss of function the expression of *lxrα* and *HMG-CoA* was increased indicating enhanced de novo cholesterol synthesis. Thus, the observed lipid accumulation may not only result from a sequestration of cholesterol transport due to the NPC1 mutation but also from an increase of lipogenesis due to energy deprivation, which in consequence leads to impaired liver function. Even more, the *Npc1^−/−^* hepatocytes were in a state of cholesterol deficit and unable to generate ketone bodies—an alternative energy source—to improve energy balance [33].

PPARα functions as a sensor for free fatty acids. It regulates the lipid metabolism by inducing lipolysis due to the increase of β-oxidation in peroxisomes. This leads to a decrease of systemic triglycerides and hepatic cholesterol concentrations [34]. Thus, *pparα* can be considered as an antagonist of the lipogenetic *lxrα*. The activation of *pparα* increases *abca1* gene expression [35], which seems to be mediated by elevation of *lxrα* gene activation [36]. In the present study, the expression of *pparα* and of *abca1* was lowered although *lxrα* was almost unchanged. This may indicate that lipolysis was decreased in *Npc1^−/−^* mice independently of the lipogenetic *lxrα* expression. This would suggest that a loss of NPC1 function causes a shift of fat metabolism towards a lowered lipolyis which, in consequence, may enhance parameters of lipogenesis.

In the current study, a combination therapy with HPβCD/ALLO/miglustat rescued the NPC1 mutant-related dysbalance of fat metabolism. mRNA expression of *acat1* is upregulated upon both therapeutic strategies. Since it is known that *acat1* esterified cholesterol for lipid transport [5], this finding would contribute to the recovery of cholesterol esterification as described by Sakashita et al. [6]. Furthermore, the combination therapy induced a rise of *pparα* together with *acox1* and *abca1* as well as its partner *apoE* leading to increased lipolysis as well as β-oxidation and cholesterol transport. Also, monotherapy with HPβCD slowed the progression of the NPC1 transgene effects, consistent with findings from previous studies [22,37]. The treatment with HPβCD increased the mRNA expression of *pparα* and of *abca1* significantly, but the rise was more pronounced following combination of HPβCD with ALLO and miglustat. The effect of HPβCD monotherapy agreed with the observation of Taylor et al. [32] that hepatic cholesterol transporter *abca1* was upregulated in mono-treated *Npc1^−/−^* mice. Likewise, the expression of a further cholesterol transporter gene, *abcg1,* was reported to increase following HPβCD therapy, which assumingly excreted hepatic cholesterol as bile acid in treated *Npc1^−/−^* mice [38]. However, the mRNA expression of *apoE* decreased significantly following monotherapy. Since it is known that *apoE* and *abca1* cooperate in a concerted effort to mobilize cholesterol for the transport [39], one can speculate that the rescued fat metabolism upon monotherapy is rather a result of increased lipolysis followed by β-oxidation of fatty acids than cholesterol transport. This assumption is supported by the finding that mRNA expression of *acox1* is much more upregulated upon monotherapy than combination therapy. However, mRNA expression of *fatp2* is found to be strongly upregulated upon monotherapy. Therefore, it can be assumed that HPβCD restores lipid homeostasis beside intensified lipolysis or β-oxidation also via the transport of fatty acid which in turn is the precondition of increased β-oxidation of fatty acids. Although *srebp-2* stimulates cholesterol synthesis [40], its expression was found to be increased upon combination therapy. Since *srebp-2* positively regulates transcription of *abca1* by generating oxysterol ligands for *lxrα* [41], it can be assumed that upregulated *srebp-2* expression upon combination therapy might be responsible for lipid efflux rather than for cholesterol synthesis. The assumption that the combination therapy causes an enhanced lipid efflux is supported by a significant increase of plasma triglyceride concentration. In contrast to the study of Liu et al. [18] showing a downregulation of both *srebp-2* and *HMG-CoA* hepatic mRNA expression following HPβCD monotherapy, we found a strong upregulation of *srebp-2* and *HMG-CoA* mRNA expressions after HPβCD monotherapy. A potential reason for this discrepancy might be the rather long-term application of HPβCD (current study) which raises the de novo cholesterol synthesis (lipogenesis) compared to the single application of HPβCD, as reported by Liu et al. [18]. However, increased mRNA expression of *srebp-2* and *HMG-CoA* does not fit with the results of decreased hepatic cholesterol content. It is known that high proliferation rate correlates with increased cholesterol synthesis, as indicated by a high level of *HMG-CoA* [42]. Therefore, it can be assumed that increased lipogenesis upon monotherapy as shown in the current study is a result of long-term HPβCD treatment enhanced proliferation. Furthermore, it can be expected that an increase of lipogenesis causes an increase of the cholesterol content. However, mono-treated mice revealed decreased content of hepatic cholesterol. Based on the fact that a high proliferation rate, as shown upon HPβCD treatment, is an energy-intensive process [26,27] we assumed that hepatic cholesterol is used for proliferation. On the other hand, the combination therapy caused no changes in the mRNA expression of *HMG-CoA* indicating that combination therapy vs. monotherapy primarily targeted the sequestration of cholesterol by enhancing of cholesterol efflux.

Furthermore, it has been reported that cholesterol efflux from mouse macrophages (foam cells) is associated with the cholesterol transporter and lipolytic genes *pparα*, *lxrα*, *abca1* and *apoE* [43]. Therefore, it is conceivable that besides diminishing hepatic cholesterol accumulation upon monotherapy and combination therapy, the decrease of macrophage (foam cell) numbers in sinusoids is triggered by cholesterol transporter and lipolytic genes, e.g., *pparα* and *abca1.* The removal of cholesterol leads to a reduction of apoptotic cell death and, therefore, to a better morphology and function of the liver. This interpretation agrees with the studies of Liu et al. [18] and Tanaka et al. [19] who showed the hepatoprotective effect of a single dose monotherapy with HPβCD in *Npc1^−/−^* mice. Nevertheless, cytotoxic effects of HPβCD at a relatively high dosage are possible, but Tanaka et al. [19] showed that HPβCD applied with a dosage of 4000 mg/kg in *Npc1^−/−^* mice causes the best survival rate, when compared to saline and to other, in particular smaller, dosages. However, it is also described that upon HPβCD treatment with a dosage of 4000 mg/kg in *Npc1^−/−^* mice the cholesterol synthesis is decreased [44], but to the value observed in the *Npc1^+/+^* mice [23]. Therefore, the used dosage of HPβCD may cause no changes of cholesterol synthesis in a pathological manner.

In summary, both the combination therapy and monotherapy caused a reduction of hepatic lipids, while the monotherapy mainly decreased hepatic cholesterol content. Nevertheless, both therapeutic strategies ameliorated NPC1 liver disease symptoms. The effect of the monotherapy directed *pparα*- and *acox1*-associated lipolysis/β-oxidation, whereas the combination therapy increased not only the lipolysis but also the cholesterol transport via *abca1* and *apoE*. Moreover, the monotherapy caused an increase of fatty acid transport. However, HPβCD monotherapy additionally increased the cholesterol synthesis, probably as a result of increased hepatocellular proliferation or vice versa.

The novelty of this comparative study is that the combination therapy acts via an increase of hepatic cholesterol transport and lipolysis, whereas monotherapy operates via increased hepatic fatty acid transport and β-oxidation. Lipolysis is also activated, but not to this extent, as observed upon combination therapy.

## 4. Methods

### 4.1. Animals

Mice were kept under standard laboratory conditions (12 h light/dark cycle; 55 ± 15% humidity; 24 ± 2 °C room temperature (RT) and water ad libitum) in accordance with German and European guidelines (2010/63/EU) for the use of laboratory animals. Approval of experiments was obtained from the local Committee on the Ethics of Animal Experiments of Mecklenburg Vorpommern (7221.3-1.1-088/10; date of approval: 22 March 2011; 7221.3-1-30/12; date of approval: 14 June 2012; 7221.3-1-011/16; date of approval: 4 May 2016). Heterozygous breeding pairs of BALB/cNctr-Npc1m1N/J (*Npc1^−/−^*) mice were obtained from the Jackson Laboratories (Bar Harbor, ME, USA) for generating homozygous *Npc1^−/−^* mutants and control wild type mice (*Npc1^+/+^*).

### 4.2. Pharmacological Treatment

**Figure 10:**
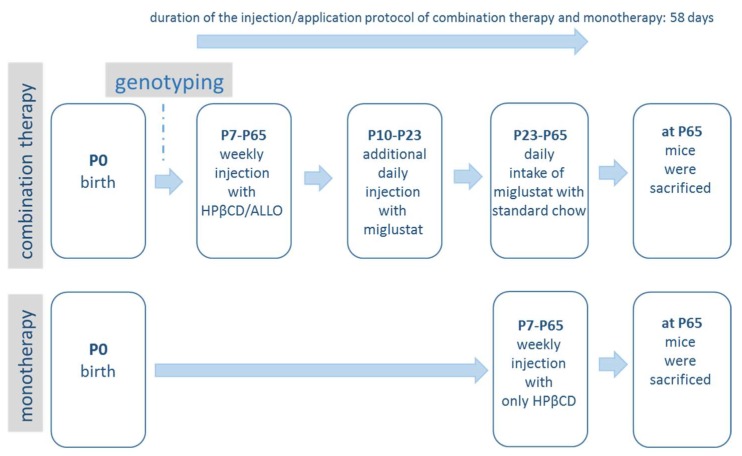
Scheme of the drug application for the combination therapy and monotherapy (ALLO, allopregnanolone; HPβCD, 2-hydroxypropyl-β-cyclodextrin).

The control mice received either sham treatment, further referred to as sham-treated controls (up to *n* = 16), or received the combination therapy and are further referred to as combi-treated controls (up to *n* = 18). The mutant group (*Npc1^−/−^*) was also divided in subgroups, receiving either sham (sham-treated; up to *n* = 16) or treatment of combined therapy (combi-treated, up to *n* = 11). An additional group of each *Npc1^−/−^* (*n* = 15) and *Npc1^+/+^* (*n =* 11) mice received only HPβCD and are further referred to as mono-treated mice. The injection protocol is shown in Figure 10. Starting at postnatal day 7 (P7) and thenceforth, mice of the combi group were injected weekly with HPβCD/ALLO (25 mg/kg ALLO dissolved in 40% HPβCD in Ringer’s solution, 4000 mg/kg, intraperitoneal (i.p.), HPβCD solubilizes ALLO and serves as the vehicle for ALLO; all from Sigma-Aldrich, Munich, Germany). Additionally, 300 mg/kg miglustat (*N*-butyldeoxynojirimycin, Zavesca^®^; a generous gift of Actelion Pharmaceuticals, Allschwil, Switzerland) dissolved in 0.9% NaCl solution were daily injected from P10 to P23. From P23 onwards until termination of experiments mice were fed standard chow with embedded miglustat resulting in daily intake of 1200 mg/kg miglustat. The mice receiving only HPβCD were injected weekly with HPβCD (4000 mg/kg in Ringer’s solution, i.p. Sigma-Aldrich) starting at P7. As previously described by our group [45] mice of the sham group were injected like those of the combi group at the various time points with respective volumes of 0.9% NaCl or Ringer’s solution, and were fed with chow without drugs. The current dosage regimen was chosen in accordance to previously published studies [20,21,45,46]. In addition, Tanaka et al. [19] showed that HPβCD applied at a dose of 4000 mg/kg in *Npc1^−/−^* mice caused the best survival rate when compared to saline.

### 4.3. BrdU Injections

To label proliferating cells, an additional group of mice were intraperitoneally injected with 5-bromo-2′-deoxyuridine (BrdU, solubilized in 0.9% NaCl solution, 50 mg/kg, Sigma-Aldrich) twice a day from P40 to P46. Additionally a final single dose was given 1 h before perfusion at P55–56.

### 4.4. Sampling and Assays

All mice were deeply anaesthetized with an overdose of pentobarbital, weighed and exsanguinated by puncture of the vena cava inferior for immediate separation of plasma, followed by harvest of non-perfused liver tissue. The livers were weighed. Aspartate aminotransferase (AST) and alanine aminotransferase (ALT) activities were measured spectrophotometrically as indicators for hepatocellular disintegration and necrosis. The extinction at 340/378 nm was measured with the cobas^®^c111 Analyzer (Roche Diagnostics GmbH, Penzberg, Germany). Measurements of plasma triglycerides and ketone bodies were performed using the assay kit methods according to the manufacturer’s instructions (Cayman Chemical Company, Hamburg, Germany). For measurements of the plasma apoE concentration, plasma was diluted 1:500 and instructions of the manufacturers were followed (LifeSpan Bioscience, Inc., Seattle, WA, USA).

For measurement of hepatic cholesterol concentrations, lipids were extracted by means of the Bligh-Dyer method [47] and described by our group [48]. Livers were incubated with a mixture of chloroform and methanol (1:2). After vortexing one part of chloroform and one part of H_2_O was added and separated into two phases by centrifugation at 3000× *g*. As previously described by our group [48] the organic phase (lower layer) was collected and concentrated by vacuum pump. According to the manufacturer’s instructions (Calbiochem, Merck KGaA, Darmstadt, Germany) the cholesterol content was analyzed by using the cholesterol/cholesteryl ester quantitation kit method.

### 4.5. Quantitative Real-Time RT-PCR Analysis

As previously described by our group [48] and in accordance with the manufacturer’s instructions total RNA was isolated using the RNeasy^®^ Mini Kit (Qiagen, Hilden, Germany). As described by the manufacturer 2 µg of total RNA was reverse-transcribed with SuperScript^TM^ First Strand Synthese System (ThermoFisher Scientific, Waltham, MA, USA). By using Lightcycler 1.5 and Lightcycler^®^ FastStart DNA MasterPlus SYBR Green I kit (Roche Diagnostics GmbH) real-time quantitative PCR assays were performed. As described previously by our group [48] each amplification mixture (20 µL) contained 5 µM primer, 19 µL of universal PCR Mastermix (Roche Diagnostics GmbH), and 1 µL 1:2 diluted cDNA solution. PCR thermocycling parameters were 95 °C for 10 min and 40 cycles of 95 °C for 10 s, 55 °C for 5 s and 72 °C for 10 s. All samples were analyzed for *cyclophilin A* (*ppia*) expression [49]. Using the 2^−ΔΔ*C*t^ method the relative change in gene expression were analyzed. As the control and therefore as the first Δ served a cDNA pool of livers of sham-treated mice. The second Δ is represented by *C*_t_-values of *cyclophilin A* (*ppia*) amplification. Specificity of the amplification was verified by melt-curve analysis and evaluation of efficiency of PCR amplification. The primers are listed in Table 1.

### 4.6. Histology and Immunohistochemistry

Liver tissue was fixed in 4% phosphate-buffered formalin for 2–3 days, embedded in paraffin, sectioned at 5 µm thickness, mounted on poly-l-lysine covered glass slides and stained with hematoxylin and eosin (H&E). With the AS-D chloroacetate esterase (CAE) technique granulocytes were stained and identified by positive staining and morphology within the granulation tissue [48]. For immunohistochemical analysis of cleaved caspase-3^+^ cells, F4/80^+^ von Kupffer cells (resident liver macrophages) and BrdU^+^ hepatocytes sections were pre-treated with citrate puffer (pH 6.0) in the microwave (700 W for 7 min) and were either exposed to a rabbit anti-cleaved caspase-3 antibody (1:500, Cell Signaling Technology, Frankfurt, Germany), a rat anti-F4/80 (1:10; Serotec, Oxford, UK) or a mouse anti-BrdU antiserum (1:50; DakoCytomation, Dako, Hamburg, Germany). In accordance to the manufacturer’s instructions the 3,3′-diaminobenzidine (DAB) chromogen Universal LSAB^®^ kits (System-HRP; DakoCytomation) were used for the development of the primary antibodies. As previously described by our group [48] the sections were counterstained with hemalaun, cover slipped and analyzed with a light microscope (Olympus BX51, Hamburg, Germany). Images were acquired with a Color View II FW camera (Color View, Munich, Germany) and adjusted in brightness and contrast with Photoshop CS2 software (Adobe Systems, Version 12.0, San Jose, CA, USA). CAE^+^, F4/80^+^ cells and BrdU^+^ hepatocytes were counted within 50 consecutive fields of liver tissue (×40 objective) and are given as cells per high power field (HPF).

### 4.7. Electron Microscopy

Each two animals of sham-treated *Npc1^+/+^*, *Npc1^−/−^* and combi-treated *Npc1^−/−^* were killed by an overdose of sodium pentobarbital. Then, cardiac perfusion with phosphate-buffered saline (PBS, pH = 7.4) was followed by 4% paraformaldehyde (PFA) in 0.1 M PBS. As previously described by our group [50] after perfusion and preparation liver samples were excised and postfixed in 0.1 M phosphate buffer containing 2.5% glutaraldehyde for at least 24 h at 4 °C. Thereafter, the specimens were osmicated, washed, block contrasted with 2% aqueous uranyl acetate, dehydrated through a graded series of ethanol, and embedded in Epon 812 (Plano GmbH, Marburg, Germany). As previously described by our group [50] ultrathin sections (about 70 nm) were mounted on pioloform-coated slot copper grids and contrasted with uranyl acetate (4 min) followed by lead citrate (2 min). The specimens were examined with a Zeiss EM 902 transmission electron microscope (Zeiss, Oberkochen, Germany) at 80 kV. Electron micrographs were taken using a 1 × 2 FT-CCD camera (Proscan, Scheuring, Germany) and adjusted using Photoshop CS2 software (Adobe Systems).

### 4.8. Statistical Analysis

As previously described by our group [48] all data are expressed as means ± SEM. Statistical differences were determined using ANOVA, followed by *post-hoc* pairwise comparison tests for analysis. Data were considered significant if *p* < 0.05. Statistical analysis was performed using the Sigma Stat and Sigma Plot software package (version 12.0, Jandel Scientific, San Rafael, CA, USA).

## Figures and Tables

**Table 1:** List of Primers used for RT-PCR.

Transcript	Forward Primer (5′–3′)	Reverse Primer (5′–3′)
*abca1*	ACT GGA GAC ACC CCT GTG AC	GGA GAG CTT TCG TTT GTT GC
*apoE*	GTC TGA CCA GGT CCA GGA AG	AGT CGG TTG CGT AGA TCC TC
*fas*	TAC CAT GGC AAC GTG ACA CT	TAG CCC TCC CGT ACA CTC AC
*HMG-CoA*	CAG GAT GCA GCA CAG AAT GT	CTT TGC ATG CTC CTT GAA CA
*lxrα*	CAC CGC CAA ATT TAA CTG CAG A	AAG GGT TTG ATA AGT TCT AGC TGT
*pparα*	GGA AGC CGT TCT GTG ACA TC	TCA TCT GGA TGG TTG CTC TG
*ppia*	ACC AAA CAC AAA CGG TTC CC	CCA CAG TCG GAA ATG GTG ATC
*srebp-1c*	GAT CGC AGT CTG AGG AGG AG	GAT CGC CAA GCT TCT CTA CG
*srebp-2*	ACC TGT GAC CTG CTA CTG TC	CAG CTG GTG TGT ACG GGT AG
*acox1*	GAG CTG CTC ACA GTG AC TCG	ACT GCA GGG GCT TCA AGT G
*acat1*	AGC ATT CAG TGT GGT TGT GC	TCC TCC TCC GTT GCA AAT AC
*fatp2*	AAC ACA TCG CGG AGT ACC TG	CTC AGT CAT GGG CAC AAA TG
